# 39.4 A DOUBLE-BLIND TRIAL OF VALACYCLOVIR TO IMPROVE COGNITION IN EARLY PHASE SCHIZOPHRENIA: RESULTS FROM THE VISTA STUDY

**DOI:** 10.1093/schbul/sby014.162

**Published:** 2018-04-01

**Authors:** Alan Breier, Faith Dickerson, Robert Buchanan, Stephen Marder, Keith Neuchterlein, Deepak D’Souza, Michael Francis, Alexander Radnovich, Robert Yolken, Sheldon Preskorn, Matthew Macaluso, Ziyi Yang, Nicole Mehdyoun, Rishi Kakar, Walter Dunn, Debra Hoffmeyer, Gerald Maguire

**Affiliations:** 1 Indiana University School of Medicine; 2 Sheppard Pratt; 3 Maryland Psychiatric Research Center; 4 University of California, Los Angeles; 5 Yale University School of Medicine, VA Connecticut Healthcare System; 6 Johns Hopkins University School of Medicine; 7 Kansas University Medical Center; 8 Segal Trials; 9 CI Trials; 10 University of California, Riverside

## Abstract

**Background:**

Several lines of evidence suggest that Herpes Simplex Virus type 1 (HSV-1) may contribute to cognitive impairment in schizophrenia. Herpes viruses are enveloped, double stranded DNA viruses that are capable of infecting human CNS resulting in life-long infection with over 40% of the population seropositive for this virus. Valacyclovir is an effective treatment for the suppression of herpes virus infections. A previous small clinical trial (N=24) assessed the efficacy of valacyclovir for cognitive impairment in patients with early phase schizophrenia who were seropositive for HSV-1 (Prasad et al 2013). Results indicate that adjunctive valacyclovir, as compared to placebo, showed improvement in working and visual memory. The current study was undertaken to confirm and extend these findings and determine if HSV-1 seropositive, as compared to those who were HSV-1 seronegative, derived cognitive improvement from adjunctive valacyclovir. We hypothesized that individuals who were HSV-1 positive, but not HSV-1 negative, would demonstrate significant valacyclovir efficacy for cognitive impairment.

**Methods:**

An early psychosis network comprised of 12 US sites was established for the valacyclovir trial. Subjects had early phase schizophrenia (within 8 years since psychosis onset) and were randomized 1:1 to a 16-week trial of adjunctive valacyclovir (3 grams/day) or placebo. Assessments included cognitive domains (MATRICS), role functioning (UPSA-B, Q-LES-Q-SF, PSP), psychiatric symptoms (PANSS, NSA-16) and a range of inflammatory markers. The primary outcome was change in working (composite score of Spatial Span and Letter Number span) and visual (Brief Visuospatial Memory Test) as measured by the MATRICS.

**Results:**

170 subjects with early phase schizophrenia were stratified by HSV-1 status and randomized 1:1 to adjunctive valacyclovir or adjunctive placebo. Of those randomized, 74 were HSV-1 seropositive and 96 were seronegative. The valacyclovir vs. placebo groups, respectively, were well matched: age (28.4 vs. 27.5 years), gender (males: 69% vs. 77.9 %), race (white-caucasian: 28.6% vs. 33.7%) and duration of psychosis (3.7 vs. 4.0, years). Baseline working memory scores (Letter-Number Span) were significantly lower in HSV1 positive vs. negative subjects (mean/SD: vs. 35.1/9.0 vs 38.3/11.0, p=0.046). Treatment outcome analyses have only recently commenced and are ongoing. Full results of the cognitive, functioning, symptom and safety measures will be presented at the meeting.

**Discussion:**

The current study included 170 patients with early phase schizophrenia randomized to valacyclovir or placebo and stratified by HSV1 sero-status. Baseline working memory scores (Letter-Number Span) were significantly lower in HSV1 positive compared to HSV1 negative subjects. As analyses of treatment effects are ongoing, detailed results of valacyclovir’s effects on cognitive domains, symptoms, functioning and safety will be presented at the meeting. Additional research is needed to determine the full therapeutic potential of valacyclovir and other medications targeting herpes viruses in the treatment of schizophrenia.

